# Association Between Vaquejada Practice and Biomarkers of Oxidative Stress, Inflammation, and Muscle Damage in Quarter Horses

**DOI:** 10.3390/vetsci13060531

**Published:** 2026-05-29

**Authors:** Rayane Caroline Medeiros do Nascimento, Erik Antônio Barros Guedes, Rafael Barbosa da Silva, Islany Thaissa Izidoro Cerqueira, Cláudio Cesár dos Santos Freire, Diogo Ribeiro Câmara, Fabiana Andrea Moura, Pierre Barnabé Escodro, Marília Oliveira Fonseca Goulart

**Affiliations:** 1 Institute of Chemistry and Biotechnology, Federal University of Alagoas (UFAL), Maceió 57072-900, AL, Brazilmofg@qui.ufal.br (M.O.F.G.); 2 Campus of Engineering and Agricultural Sciences, Federal University of Alagoas (UFAL), Viçosa Campus, Viçosa 57700-000, AL, Brazildiogo@vicosa.ufal.br (D.R.C.);; 3 Faculty of Nutrition, Federal University of Alagoas (UFAL), Maceió 57072-900, AL, Brazil; fabiana.moura@fanut.ufal.br

**Keywords:** athletic performance, redox imbalance, equine exercise physiology, lipid peroxidation

## Abstract

Vaquejada (VQ) is a traditional Brazilian sport demanding high performance from Quarter Horse (QH) athletes. This study aimed to identify modifications in hematological, biochemical, inflammatory, and oxidative stress biomarkers associated with VQ training, comparing conditioned and non-conditioned horses. Blood samples were collected at rest from 70 healthy QH horses: 60 athletes (VQ-conditioned athlete group—AG) and 10 sedentary controls (breeding horses—CG). Using Linear Mixed Models alongside comprehensive hematological and biochemical analyses, the study found that AG horses exhibited an altered homeostatic profile, characterized by significant reductions in erythrocytes (7.0 [2.7] vs. 8.0 [1.6]; *p* = 0.021), lymphocytes (33.0 [21.0] vs. 41.0 [25.0]; *p* = 0.028), and the antioxidant enzymes catalase (1.5 [0.5] vs. 2.4 [1.4]; *p* = 0.026) and myeloperoxidase (141.5 [53.9] vs. 235.6 [30.4]; *p* = 0.022) relative to CG. Conversely, marked increases were observed in band neutrophils (59.0 [16.0] vs. 49.0 [27.0]; *p* = 0.028), platelets (276.0 [115.0] vs. 160.0 [113.6]; *p* = 0.026), and malondialdehyde levels (177.5 [211.8] vs. 1475.0 [802.5]; *p* < 0.001), evidencing lipid peroxidation. These findings indicate that VQ induces severe oxidative stress and compromises immune functions, with horses presenting oxidative rather than inflammatory damage. Dietary monitoring is therefore recommended to mitigate such effects at 96 h post-exercise.

## Simple Summary

Vaquejada (VQ) is a traditional equestrian sport from Northeastern Brazil, typically performed by Quarter Horses (QH), characterized as a high-intensity, short-duration physical activity. This practice can induce temporary or permanent stress, injuries, and muscular damage in horses. This study compared resting QH athletes with non-athletes to better understand the effects of VQ conditioning on biomarkers associated with physical exercise. Results indicated that athletes exhibited stronger evidence of alterations in coagulation and oxidative stress markers, despite showing no significant changes in inflammatory responses. These findings demonstrate that intense exercise can directly impact equine health. The research underscores the importance of monitoring animal welfare and suggests implementing strategies to mitigate the oxidative effects of this short, intense physical effort.

## 1. Introduction

Oxidative stress (OS) is characterized by an imbalance between pro-oxidant and antioxidant systems that may result in damage to biological systems, depending on concentration and contextual factors [1,2]. During physical exercise, increased oxygen demand by muscles intensifies the generation of reactive oxygen species (ROS) [3]. When produced at physiological concentrations, ROS play a pivotal role in redox signaling, modulating adaptive processes such as muscle contraction, mitochondrial biogenesis, and activation of the endogenous antioxidant response—a state referred to as oxidative eustress [2,4]. However, when ROS production exceeds antioxidant capacity, as observed during exhaustive exercise or adverse metabolic conditions, OS occurs, leading to disrupted redox signaling, molecular damage, and inflammation—a process termed distress [1,2,5,6].

OS promotes the accumulation of ROS and reactive nitrogen species (RNS), which interact with essential molecular, cellular, and tissue structures, including lipid membranes, proteins, and nucleic acids [7]. Beyond direct oxidative damage, an amplified inflammatory response arising from muscle injury and the subsequent tissue repair process may predispose athletes to recurrent lesions and compromised performance [8,9]. Measuring serum biomarkers from multiple sources—including antioxidant enzymes, low-molecular-weight (LMW) antioxidants, and oxidation products such as malondialdehyde, catalase, superoxide dismutase, hydrogen peroxide, and myeloperoxidase—represents an effective strategy for assessing OS and inflammation in equine athletes [10]. Such monitoring, combined with interventions designed to counteract harmful effects, supports the maintenance of high performance and athletic longevity in horses [11].

In the context of equine exercise physiology, the Vaquejada (VQ) is a traditional sport from Northeastern Brazil in which horses must accelerate rapidly and reach high speeds over short distances. The vaquejada consists of the coordinated action of two horse-rider binomials responsible for guiding a bovine along a sand track measuring approximately 90 to 150 m. The maneuver culminates in the grounding of the animal within a demarcated 10 m zone. This demand makes the Quarter Horse (QH) breed particularly suited to the exercise, as it is also widely employed in other modalities, including roping, reining, and barrel racing [5,9,11]. Nevertheless, information on the effects of QH training for VQ on redox status and inflammatory biomarkers remains limited [7]. The present study, therefore, aimed to evaluate the effects of VQ training on hematological, biochemical, oxidative, and inflammatory profiles of horses assessed at rest, compared with non-athlete QH used as controls.

## 2. Materials and Methods

### 2.1. Ethical Aspects

This research involved VQ-trained athlete horses (conditioned for sport and in the competition phase) and sedentary QH (originating from a breeding center) from Alagoas, Brazil. All procedures were approved by the Ethics Committee on Animal Use of the Federal University of Alagoas (CEUA-UFAL) under protocol #01/2024. Written informed consent was obtained from all owners prior to participation.

### 2.2. Animals and Experimental Design

This observational, cross-sectional, comparative study allocated QH into two groups: VQ-trained athletes (Athlete Group—AG) and non-athletes (Control Group—CG). Data collection occurred between September and October 2024 to minimize the influence of environmental variables. Animals belonged to four studs located within the same geographical region. All subjects were maintained under an intensive management system; Dietary regimen consisted of Tifton 85 hay, mineral salt, and water ad libitum, supplemented with pelleted feed at 1% body weight, administered three times daily, with group-specific formulations.

### 2.3. Inclusion and Exclusion Criteria

The study enrolled 70 QH, comprising 60 VQ-trained animals (AG) and 10 non-athletes (CG). Although sex distribution was not proportional across groups, the primary variable of interest was athletic activity, not sex, which was included as a fixed factor in the statistical model.

General inclusion criteria required horses aged 4–10 years, weighing 350–550 kg, clinically healthy, and presenting satisfactory scores on the EQUIBEA-UFAL welfare scale adapted from [12]. Health and welfare assessments encompassed clinical parameters, blood sampling, and owner-completed questionnaires.

Inclusion Criteria for Vaquejada Athlete (AG) Classification: Participants must actively practice one of the four disciplines that constitute the sport of Vaquejada:

1. According modality is stratified by age horses (initial horse levels):
  (a) Foal (Colt/Filly) category (animals up to 5 equestrian years);
  (b) Derby category (animals up to 7 equestrian years);
2. According to difficulty and technical proficiency (horse-rider level):
  (a) Amateur category (experienced horse and less experienced rider);
  (b) Professional category (high-performance binomials with advanced technical expertise)—professional categories compete with cattle of greater body mass;

Inclusion Criteria for Non-Athlete Classification: Not performing physical exercises of training or competition for at least 12 months. CG animals were used exclusively for breeding.

General Exclusion Criteria:

(b) Pregnancy/Gestation;
(c) Lactation/Postpartum period < 6 months;
(d) Current use (as per stud farm records) of medications such as antibiotics, anticoagulants, antiplatelets, hormones, and anti-inflammatory drugs;
(e) Post-surgical recovery < 3 months.
(f) For AG horses—any interruption in physical activity.

### 2.4. Sample Collection and Processing

All samples were collected from horses at rest in their stables in the morning, between 08:00 and 10:00 h. For AG animals, collection was performed 96 h after competition and prior to transport to the subsequent event. Blood was obtained by jugular venipuncture using EDTA tubes (BD Vacutainer^®^, Franklin Lakes, NJ, USA) for hematological, inflammatory, and OS marker analyses, including total plasma protein (TPP) and fibrinogen. Clot activator tubes with separator gel (Vacutainer^®^, Franklin Lakes, NJ, USA) were used for biochemical analyses. Samples were immediately refrigerated and transported to the laboratory for processing.

The 96 h window is a standard interval used by trainers to allow full recovery before resuming training. Blood sampling from CG horses was performed on a single day, since all animals were housed in the same stud. For AG horses, a minimum post-competition interval of 96 h was strictly observed prior to collection. This standardized recovery period was implemented to ensure normalization of acute exercise-induced fluctuations in hematological, biochemical, oxidative, and inflammatory markers, thereby reflecting the chronic, stabilized physiological status of animals at rest.

#### 2.4.1. Hematological and Biochemical Analyses

Hematological analysis was performed using an automated analyzer (BC-2800Vet, Mindray^®^, Shenzhen, China) to determine complete blood count (erythrogram and leukogram) and TPP. Platelet counts and leukocyte differentials were assessed by optical microscopy [13], and TPP was confirmed by refractometry. Plasma samples were obtained by centrifugation (4 °C, 4000 rpm, 10 min), transferred to microtubes, and stored at −80 °C. Serum samples were obtained by centrifugation (4000 rpm, room temperature, 5 min), aliquoted, and stored at −20 °C.

Plasma fibrinogen (mg/dL) was measured by the heat precipitation method at 56 °C in microhematocrit tubes [14]. Serum concentrations of creatine kinase (CK, U/L), aspartate aminotransferase (AST, U/L), and gamma-glutamyl transferase (GGT, U/L) were determined using an automated biochemical analyzer (Max Bio 300, Medmax^®^, São Gonçalo, Rio de Janeiro, Brazil) through colorimetric kinetic assays (BioClin Quibasa^®^ kits, Belo Horizonte, Minas Gerais, Brazil) [15]. Cortisol levels were quantified by chemiluminescence (Immulite^®^ 1000 Systems, Siemens Healthineers^®^, Erlangen, Germany).

#### 2.4.2. Oxidative Stress and Cytokine Biomarkers

All OS and inflammation markers were analyzed in duplicate at controlled temperatures (20–22 °C) using calibration curves (R^2^ ≥ 0.98). All spectrophotometric readings were performed on a Tecan Infinite 200 Pro (Tecan Austria GmbH, Grödig, Austria).

Malondialdehyde (MDA, pmol/mL) was quantified by High-Performance Liquid Chromatography (HPLC) (Shimadzu Corporation^®^, Kyoto, Japan) with UV detection at 270 nm, using a C18 silica column and a mobile phase of acetonitrile and Trizma buffer (pH 7.4) at a 1:9 ratio [16]. Samples prepared with plasma, Trizma, butylated hydroxytoluene, and acetonitrile were centrifuged (3500 rpm, 10 min, 4 °C), filtered through 0.22 µm membranes, and injected at 20 µL.

Superoxide dismutase (SOD, U/mg TPP) activity was determined spectrophotometrically using a commercial assay kit (Sigma-Aldrich^®^, St. Louis, MO, USA) at 450 nm. Catalase (CAT, U/mg TPP) activity was measured at 374 nm using an adapted method [17]. Hydrogen peroxide (H_2_O_2_) concentration was determined by a colorimetric method based on phenol red oxidation mediated by horseradish peroxidase, read at 610 nm [18]. Myeloperoxidase (MPO, U/mg TPP) activity was assessed at 460 nm using an adapted method [19].

Cytokine analysis was performed by Enzyme-Linked Immunosorbent Assay (ELISA) using species-specific kits (PeproTech^®^, Cranbury, NJ, USA, validated for equine samples) for tumor necrosis factor-alpha (TNF-α), interferon-gamma (IFN-γ), interleukin-6 (IL-6), and interleukin-10 (IL-10), read at 450 nm. Cytokine concentrations were expressed as pg/mL.

### 2.5. Statistical Analysis

Statistical analyses were conducted using IBM SPSS Statistics (version 26.0). Continuous variables are reported as medians and interquartile ranges (IQR).

To assess the association between athletic status and biomarkers while accounting for the hierarchical data structure, Linear Mixed Models (LMM) were employed. Athletic status (AG vs. CG) and sex were included as fixed factors; age and body weight were included as covariates. The interaction between athletic status and sex was also tested. The stud farm was included as a random intercept to account for potential clustering effects. To control the false discovery rate (FDR) arising from multiple comparisons (m = 28 biomarkers), the Benjamini–Hochberg (BH) procedure was applied, with significance defined at an adjusted *p*-value (*q*-value) < 0.20. For all remaining analyses, the significance threshold was set at *p* < 0.05.

To examine relationships among biomarkers that remained significant after LMM, a Pearson correlation heatmap was generated. Correlation strength was classified as weak (*r* = 0.20–0.39), moderate (*r* = 0.40–0.69), or strong (*r* > 0.70).

### 2.6. Artificial Intelligence Use

**Figure 1:**
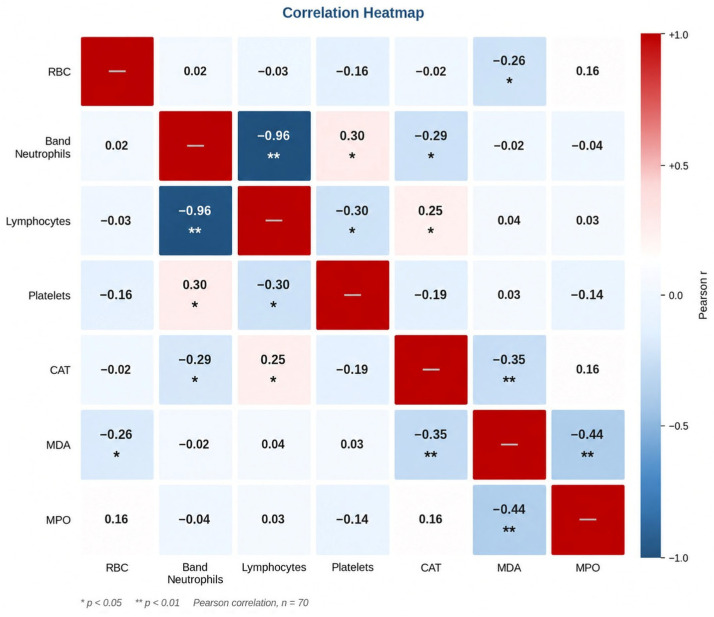
Pearson correlation heatmap of significant hematological, biochemical, and oxidative stress biomarkers in Vaquejada athletes and control horses during rest. Legend: CAT: catalase; MDA: malondialdehyde; MPO: myeloperoxidase; RBC: red blood cells. Values represent Pearson correlation coefficients (r).

**Figure 2:**
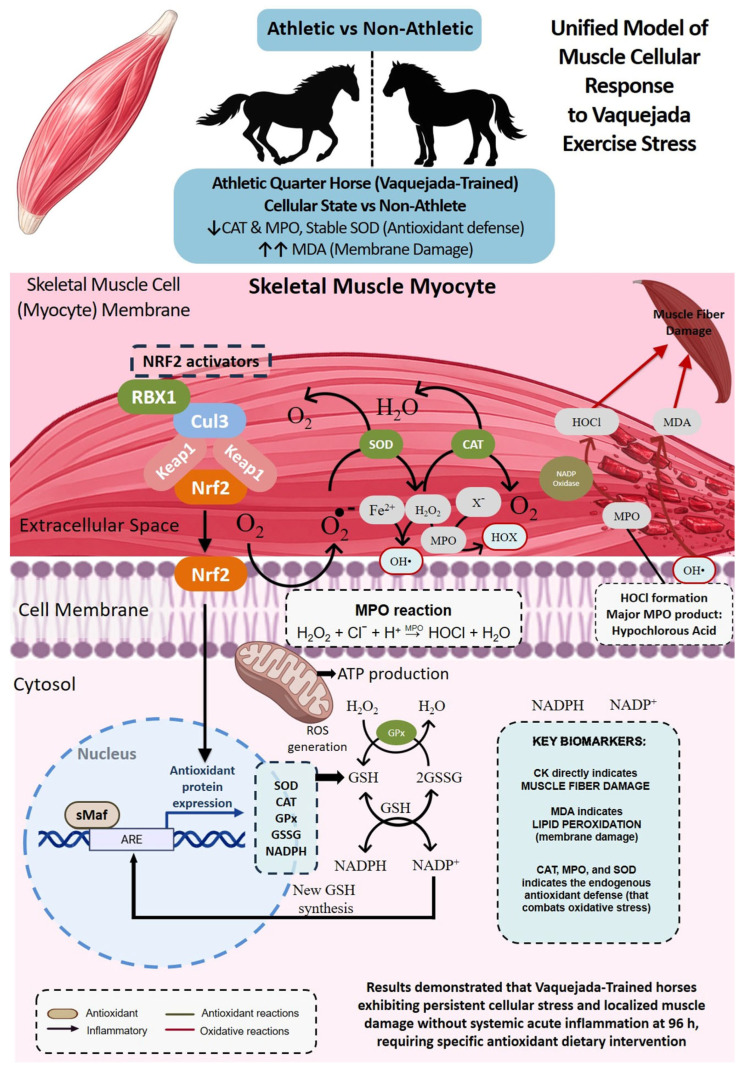
Animal ↑ Schematic development illustrating oxidative stress mechanisms and muscle inflammation in conditioned Vaquejada Quarter Horses at rest and during the post-exercise period, in comparison with sedentary horses. The athletic group exhibited lower plasma levels of MPO and CAT, while SOD activity remained stable, and MDA was at high concentrations. Legend: ↑: increase; ↓: decrease; reactive oxygen species (ROS); O_2_: singlet oxygen; O_2_•^−^: superoxide anion radical; SOD: superoxide dismutase; H_2_O_2_: hydrogen peroxide; CAT: catalase; •OH: hydroxyl radical; Fe^2+^: ferrous iron; X^−^: generic anion; MPO: myeloperoxidase; HClO: hypochlorous acid; GPx: glutathione peroxidase; GSH: reduced glutathione; GSSG: oxidized glutathione; NADPH: reduced nicotinamide adenine dinucleotide phosphate; NADP^+^: oxidized nicotinamide adenine dinucleotide phosphate; MDA: malondialdehyde; CK: creatine kinase; LH: polyunsaturated lipid; L•: lipid radical; LOOH: lipid hydroperoxide; LOOH: lipid peroxyl radical; Nrf2: nuclear factor erythroid 2-related factor 2; sMaf: small musculoaponeurotic fibrosarcoma proteins; ARE: antioxidant response element.

The following AI tools were used in this study: the Gemini 1.5 Pro model supported the design and refinement for linguistic refinement and improvement of textual clarity, as well as the standardization of bibliographic references in Vancouver style; the Claude 3.5 model was used to generate the heatmap visualization of Figure 1; Microsoft PowerPoint (2016) and BioRender (2026) were used to generate Figure 2 and Graphical Abstract.

## 3. Results

Mean age did not differ between AG and CG (6.8 ± 3.1 vs. 6.4 ± 1.5 years, respectively; *p* = 0.696). However, mean body weight was significantly greater in CG than AG (492.0 ± 50.0 vs. 452.0 ± 31.0 kg; *p* = 0.044). Sex distribution differed significantly between groups (*p* = 0.005): AG comprised 36 males and 24 females, whereas CG included 1 male and 9 females. This disparity reflects the reproductive function of CG animals, which are used exclusively for breeding and therefore consist predominantly of females.

In the present study, in addition to functional roles, the modality is stratified by technical proficiency (horse-rider level) and difficulty. The distribution of the studied animals across competitive categories was as follows: 10% (n = 6) belonged to the Foal (Colt/Filly) category; 5% (n = 3) were in the Derby category; 20% (n = 12) competed in the Amateur category; and 50% (n = 30) were classified in the Professional category.

The LMM revealed no significant interaction between athletic status and sex for any of the analyzed parameters (*p* > 0.05), indicating that physiological adaptations to VQ exercise were comparable across sexes, regardless of group composition.

Regarding hematological variables (Table 1), AG horses presented lower erythrocyte concentrations than CG (7.0 [2.7] vs. 8.0 [1.6]; *p* = 0.021). Band neutrophil counts were higher in AG than CG (59.0 [16.0] vs. 49.0 [27.0]; *p* = 0.028), while lymphocyte counts were lower (33.0 [21.0] vs. 41.0 [25.0]; *p* = 0.028). Platelet counts were also elevated in AG relative to CG (276.0 [115.0] vs. 160.0 [113.6]; *p* = 0.026).

No significant differences were observed in biochemical parameters between groups. In contrast, AG animals exhibited markedly greater oxidative damage, as evidenced by significantly higher MDA levels compared to CG (177.5 [211.8] vs. 1475.0 [802.5] pmol/mL; *p* < 0.001). Antioxidant defense was concurrently impaired, with lower CAT activity in AG than CG (1.5 [0.5] vs. 2.4 [1.4] U/mg TPP; *p* = 0.026). Although VQ practice had no significant effect on the cytokine profile—with both groups presenting comparable values for TNF-α, IL-6, IL-10, and IFN-γ—MPO activity was significantly reduced in AG relative to CG (141.5 [53.9] vs. 235.6 [30.4] U/mg TPP; *p* = 0.022), suggesting attenuated neutrophil and monocyte activity in conditioned animals.

Pearson correlation analysis of the biomarkers that remained significant after LMM identified an extremely strong negative correlation between segmented neutrophils and lymphocytes (r = −0.962; *p* < 0.001), consistent with a physiological stress response associated with training load. Within the oxidative profile, a moderate negative correlation was observed between MDA and MPO activity (r = −0.442; *p* < 0.01), and a weak negative correlation between MDA and CAT activity (r = −0.350; *p* < 0.01). Collectively, these associations suggest that elevated lipid peroxidation is associated with a relative decline in functional antioxidant capacity, reflecting a persistent oxidative challenge in AG animals even at rest.

## 4. Discussion

The findings of this study provide novel insight into the impact of VQ on the hematological, biochemical, oxidative, and inflammatory profiles of QH. The results demonstrate that conditioned animals, subjected to an intense competitive routine, exhibit a compromised redox balance, characterized by impaired antioxidant defense—as evidenced by reduced CAT activity—and consequent oxidative damage, reflected in elevated MDA levels. Conversely, VQ practice did not appear affect the cytokine profile and, unexpectedly, was associated with lower leukocyte enzymatic activity, specifically involving monocytes and neutrophils, as indicated by reduced MPO levels in AG animals.

### 4.1. Impact of Vaquejada on Hematological and Biochemical Profile

Contrary to expectations, AG horses did not exhibit significant alterations in red or white blood cell counts in the adjusted models. Prolonged training is typically associated with an expansion of circulating erythrocyte mass—often mediated by splenic contraction to optimize oxygen transport [20,21]—yet this erythroid response was absent in the present cohort. Although a marginal trend toward lower RBC counts and MCV was detected in unadjusted analyses, the absence of significance after controlling for sex and body weight indicates that athletic status was not the primary predictor of hematological variation in these animals.

Collectively, these findings suggest that VQ horses may have reached a hematological steady state, in which chronic adaptation to exercise supersedes the acute splenic responses typically reported in racing disciplines [20]. Such stability may further reflect a high degree of physical conditioning and a well-structured training regimen, both of which promote beneficial physiological adaptations that support performance and equine health [22,23].

In contrast, athletic status was a significant predictor of circulating platelet counts. Although some studies report a decline in platelet numbers immediately following acute exertion [24], the present results demonstrate a marked increase in AG relative to CG—consistent with observations in human athletes [25] and Thoroughbred racehorses [20,26], suggesting that regular physical training induces dynamic hematological adaptations.

The non-significance of cortisol levels and erythrocyte counts effectively excludes acute splenic contraction or immediate stress as the primary cause of thrombocytosis. Rather, the isolated increase in platelets supports a long-term adaptive response to training. According to [27], extended training periods induce platelet hyperactivity and enhanced aggregation capacity as part of a normal physiological response to conditioning, with regular exercise shown to attenuate the adverse effects of acute exertion on platelet activation—potentially establishing a hemostatic protective mechanism.

The specific demands of VQ, characterized by frequent muscular microlesions, may further contribute to this compensatory state. Elevated platelet availability may represent a systemic overcompensation to ensure efficient hemostasis and tissue repair [28], consistent with the concept of a cellular microlesion maintenance threshold that must be respected in high-performance athletes [29].

AG horses presented higher band neutrophil counts and lower lymphocyte concentrations than CG, with no significant difference in total leukocyte counts—a pattern consistent with findings reported by [30] in QH athletes during the post-competition period of Team Penning. According to [31], the neutrophilia observed in AG may reflect exercise-induced redistribution from the marginal to the circulating compartment, driven by epinephrine release, with concentrations varying according to exercise intensity before stabilizing after peak mobilization. The relative lymphopenia, in turn, may result from cortisol-mediated immunosuppression associated with chronic physiological stress imposed by strenuous exercise, compounded by transport, feed restriction, and environmental changes inherent to equine competition.

These findings, together with fibrinogen and cytokine concentrations, reinforce that AG animals do not represent chronically inflamed horses, but rather animals responding to intense exertion. They further suggest that, during this post-exercise window, these horses may present a transient reduction in immune competence—a so-called “immunological window” potentially increasing susceptibility to opportunistic infections. Based on this, targeted intervention with immunostimulants and antioxidant supplementation during the post-competition period is recommended.

No significant between-group differences were observed in biochemical markers of muscle injury (CK and AST), hepatic function (GGT), physiological stress (cortisol), or acute inflammation (fibrinogen), suggesting complete normalization by 96 h post-exercise. These findings diverge from [32,33], where significant elevations in these parameters were reported as markers of exercise-related stress. However, in [32], CK and AST values measured at 48 h post-exercise were comparable to those observed here at 96 h, suggesting a temporal recovery trajectory consistent with the present data. While the cross-sectional design limits direct intra-individual comparisons—several authors recommend using each animal’s own resting baseline as the reference [34,35]—the biochemical stability observed may also reflect a high degree of physiological conditioning. Unlike studies reporting significant post-exercise increases in CK [36], the concurrent stability of CK and AST in this cohort suggests efficient cellular recovery or superior muscular adaptation to the specific demands of VQ, indicating that the animals were metabolically restored at the time of sampling.

### 4.2. Impact of Vaquejada on Oxidative Profile

In equine athletes, physical exercise increases mitochondrial number and efficiency, elevating oxygen consumption (O_2_) and, consequently, electron leakage in the respiratory chain—thereby intensifying superoxide radical anion (O_2_•^−^) production. At moderate concentrations, ROS exert positive effects by activating signaling pathways that stimulate mitochondrial biogenesis and reinforce the antioxidant system. When production exceeds defense capacity, however, oxidative distress ensues, promoting inflammation and tissue damage [37]. Accordingly, well-conditioned athletes would be expected to exhibit upregulated antioxidant production to sustain redox homeostasis. In the present study, AG horses exhibited lower levels of antioxidant defense markers, suggesting that endogenous defenses and nutritional management in VQ horses are insufficient to counteract the oxidative burden imposed by training.

Reduced CAT activity in AG represents a key indicator of this redox imbalance. As the primary enzyme responsible for H_2_O_2_ decomposition, it exhibits diminished residual activity, suggesting extensive enzymatic recruitment to neutralize the high ROS flux generated during strenuous exertion [38]. Although H_2_O_2_ concentrations were statistically similar between groups, chronic CAT depletion likely resulted in insufficient peroxide neutralization over time [39].

Studies on horses competing in “Laço Comprido” (Long Roping)—another high-intensity, short-duration discipline—report redox homeostasis during basal evaluations, with no significant elevations in MDA nor reductions in CAT or SOD activity at rest in Criollo and QH athletes [40,41]. This suggests adequate chronic adaptation without evidence of persistent oxidative imbalance, contrasting with the pattern observed in VQ athletes.

Regarding MPO, AG animals presented lower activity than CG—an unexpected finding, given that intense exercise is known to induce neutrophil degranulation and elevated plasma MPO concentrations [42]. Two complementary mechanisms may account for this reduction. First, severe OS—confirmed by elevated MDA and reduced CAT in AG—may have driven excessive MPO consumption, exceeding endogenous replenishment capacity. Second, cortisol peaks known to occur in the post-exercise period [43] produce a characteristic stress leukogram (neutrophilia and lymphopenia), while simultaneously exerting an inhibitory effect on neutrophil function [44], reducing the synthesis and release of granular enzymes, including MPO. Recent studies on horses in the “Laço Comprido” (Long Roping) discipline—a high-intensity, short-duration activity—indicate redox homeostasis during basal evaluations. Research involving Creole and Quarter Horse athletes in this modality reports no significant elevations in basal MDA levels nor a decrease in CAT and SOD activity at rest. This suggests adequate adaptation without evidence of chronic oxidative imbalance associated with training, diverging from the parameters observed in VQ athletes [45,46].

Taken together, the elevated neutrophil counts alongside reduced MPO activity suggest impaired neutrophil functional capacity. Given that the MPO–hypochlorous acid (HOCl) pathway is essential for microbial membrane oxidation, its deficiency characterizes a state of functional immunosuppression that, combined with enzymatic exhaustion, may culminate in the post-exercise open window, heightening susceptibility of equine athletes to opportunistic infections.

The relationship between OS physiology and exercise in the AG relative to the CG is illustrated in the following figure (Figure 2).

As shown in Figure 2, AG horses appear to possess insufficient endogenous antioxidant defenses to counteract the OS generated by VQ’s intense physical demands. The marked elevation in MDA confirms active lipid peroxidation; however, the absence of cytokine alterations suggests no concurrent impairment of the inflammatory response at rest. This positions VQ distinctly among high-intensity, short-duration equestrian disciplines documented in the literature—including horse racing (Turf), barrel racing, roping, Freio de Ouro, and tent-pegging. Specifically, VQ appears more metabolically demanding during recovery than “Laço Comprido” and Turf, suggesting that current AG training regimens may be approaching overtraining or require ergogenic support, such as antioxidant supplementation with vitamin E and selenium.

VQ activity produced no significant changes in the basal inflammatory response, with TNF-α, IFN-γ, IL-6, and IL-10 remaining comparable between groups. This likely reflects both the timing of sampling and chronic athletic adaptation. Research by [47,48] demonstrates that while high-intensity exercise elicits a robust systemic inflammatory response—with marked increases in IL-6, TNF-α, and IFN-γ mRNA expression immediately post-exertion—these markers typically return to baseline within 24 h. Given that horses were sampled at rest and assessed as clinically healthy, cytokine stability was an expected outcome [48]. The absence of cytokine variation may additionally reflect enhanced metabolic efficiency and a reduced requirement for inflammatory pathway activation in well-conditioned animals [49,50]. In contrast to acute fatigue models, where a transient pro-inflammatory state is characteristic, the balanced cytokine profile observed here suggests that regular VQ training promotes immunological homeostasis, facilitating efficient cellular recovery without sustaining chronic systemic inflammation.

The correlation matrix reinforces the association between the leukocyte profile and redox imbalance in AG. The extremely strong negative correlation between segmented neutrophils and lymphocytes confirms a persistent physiological stress pattern characteristic of intense physical exertion rather than an infectious process. Within the oxidative profile, the negative correlation between MDA and MPO indicates that increased lipid peroxidation is associated with reduced neutrophil functional activity. Combined with the negative correlation between MDA and CAT, these findings suggest that the depletion of enzymatic defenses—specifically MPO and CAT—favors the redirection of H_2_O_2_ toward the Fenton reaction, generating hydroxyl radicals (•OH) that drive lipid peroxidation and account for the elevated MDA levels observed. Concurrently, reduced MPO availability compromises the HOCl pathway, potentially rendering animals susceptible to bacterial infections during the recovery period.

Gene expression analysis (qPCR) was not performed due to logistical and ethical constraints related to invasive procedures, as well as complete exhaustion of available blood samples across the extensive biomarker panel. Retrospective collections were not pursued, as they would not reflect the specific metabolic and physiological context of the original study period, potentially compromising temporal consistency and data reliability. Nonetheless, the functional enzymatic activities measured here directly reflect systemic antioxidant capacity and the phenotypic response to exercise.

The sample size imbalance between AG and CG (6:1) is an acknowledged limitation. Although this disproportion may affect statistical power, it was mitigated by using LMM—which are robust to unbalanced designs—and by maintaining a highly standardized CG to minimize baseline variance.

## 5. Conclusions

VQ-trained horses exhibited higher OS indices than controls, underscoring the physiological impact of this discipline on equine health. These findings contribute pioneering evidence highlighting the importance of accounting for the long-term consequences of redox imbalance in equine athletes. The OS identified in AG may be compounded by intensive training schedules and frequent competition, resulting in elevated ROS production that outpaces the enzymatic antioxidant response—a state with direct implications for athletic performance.

In summary, this study characterizes the OS burden to which VQ QH athletes are exposed and establishes a rationale for future preventive and diagnostic strategies targeting redox status monitoring, with the dual aim of preserving health and optimizing performance. Therefore, it is essential to evaluate oxidative stress markers before, during, and immediately after Vaquejada (VQ) and the equine diet, particularly regarding the need to enhance immunomodulatory therapy and antioxidant supplementation strategies. The goal should be to mitigate redox imbalance and achieve stable plasma levels of antioxidant enzymes between competitions. This study justifies further research into antioxidant therapy for VQ horses to optimize performance, monitor health, and extend their athletic lifespan. In summary, this research provides insight into the oxidative stress levels to which VQ equines (QH) are exposed and suggests the need for future preventive and diagnostic strategies to monitor redox status, thereby supporting performance optimization and health preservation.

## Figures and Tables

**Table 1:** Hematological, biochemical, oxidative stress, and inflammatory profiles of adult horses conditioned for Vaquejada compared to non-Athlete Quarter Horses during rest.

Variable	Non-Athlete Group (n = 10) Median (IIQ)	Athlete Group (n = 60) Median (IIQ)	LMM *p*-Value ^1^	BH-FDR *q*-Value ^2^
Hematological Variables				
RBC (cells × 10^6^/µL)	8.0 (1.6)	7.0 (2.7)	**0.021**	**0.112**
Hemoglobin (g/dL)	11.2 (2.7)	11.1 (2.1)	0.270	0.473
Hematocrit (%)	36.5 (8.0)	34.0 (7.0)	0.321	0.529
MCV (fL)	45.0 (1.3)	46.7 (4.5)	0.434	0.552
MCH (pg)	14.8 (0.4)	15.3 (1.7)	0.364	0.536
MCHC (g/dL)	33.0 (0.5)	33.0 (1.0)	0.254	0.473
Total Leukocytes (cells/mm^3^)	8650.0 (3500.0)	9000.0 (2700.0)	0.424	0.552
Band Neutrophils (%)	49.0 (27.0)	59.0 (16.0)	**0.028**	**0.112**
Lymphocytes (%)	41.0 (25.0)	33.0 (21.0)	**0.028**	**0.112**
Basophils (%)	0.5 (1,0)	0.0 (0.0)	0.227	0.473
Eosinophils (%)	3.0 (6.0)	3.0 (4.0)	0.061	0.213
Monocytes (%)	4.0 (3.0)	2.0 (3.0)	0.358	0.536
Platelets (×10^3^/µL)	160.0 (113.6)	276.0 (115.0)	**0.026**	**0.112**
Total plasma proteins (g/dL)	7.2 (0.4)	6.8 (0.6)	0.069	0.215
Biochemical variables				
Creatine kinase (U/L)	112.0 (88.0)	292.0 (202.0)	0.184	0.473
Cortisol (µg/dL)	3.0 (1.6)	2.7 (1.6)	0.998	0.998
Aspartate aminotransferase (U/L)	250.4 (55.1)	314.0 (100.9)	0.389	0.545
Gamma-glutamyl transferase (U/L)	14.2 (7.5)	13.9 (6.6)	0.778	0.870
Fibrinogen (mg/dL)	200.0 (200.0)	400.0 (300.0)	0.216	0.473
Oxidative stress & Inflammatory markers				
SOD (U/mgTPP)	7.1 (3.0)	7.2 (2.7)	0.808	0.870
H_2_O_2_ (nmol/ mgTPP )	1.7 (1.0)	1.8 (1.4)	0.768	0.870
CAT (U/mgTPP)	2.4 (1.4)	1.5 (0.5)	**0.002**	**0.028**
MDA (pmol/ mL )	177.5 (211.8)	1475.0 (802.5)	**<0.001**	**0.028**
MPO (U/mgTPP)	235.6 (30.4)	141.5 (53.9)	**0.022**	**0.112**
TNF- α (pg/mL)	49.7 (17.7)	37.5 (8.8)	0.251	0.473
IFN-y (pg/mL)	570.4 (877.7)	227.2 (291.9)	0.189	0.473
IL-6 (pg/mL)	201.3 (142.3)	117.3 (265.6)	0.954	0.989
IL-10 (pg/mL)	119.3 (278.6)	150.8 (436.7)	0.510	0.621

Legend: BH-FDR: Benjamini-Hochberg False Discovery Rate; CAT: catalase; IFN-γ: interferon-gamma; IL-6: interleukin-6; IL-10: interleukin-10; IQR: interquartile range; LMM: Linear Mixed Model; MCV: mean corpuscular volume; MCH: mean corpuscular hemoglobin; MCHC: mean corpuscular hemoglobin concentration; MDA: malondialdehyde; MPO: myeloperoxidase; ns: not significant; RBC: red blood cells; SOD: superoxide dismutase; TNF-α: tumor necrosis factor-alpha; TPP: total plasma proteins. ^1^ *p*-value from Linear Mixed Model with adjustment for confounders. ^2^ *q*-value from Benjamini–Hochberg False Discovery Rate correction (m = 28 total comparisons), *q* < 0.20: significant at exploratory threshold. Bold values indicate statistically significant differences (*p* < 0.05).

## Data Availability

The data presented in this study are available on request from the corresponding author. The data are not publicly available at this time as they represent the baseline (time zero) and pre-intervention parameters of an ongoing longitudinal study. These data will be utilized in future publications to evaluate the long-term effects of exercise and dietary interventions.
